# In Vitro and Ex Vivo Studies on the Absorption and Distribution of β-Cyclodextrin Polymer

**DOI:** 10.3390/pharmaceutics18070854

**Published:** 2026-07-14

**Authors:** Réka Révész, Akay Dogan Mengenli, Ágnes Rusznyák, Richárd Kajtár, István Lekli, Ildikó Bácskay, Ádám Haimhoffer

**Affiliations:** 1 Doctoral School of Pharmaceutical Sciences, University of Debrecen, Nagyerdei St. 98, H-4032 Debrecen, Hungary; revesz.reka@pharm.unideb.hu (R.R.); mengenli.akay.dogan@pharm.unideb.hu (A.D.M.); 2 Department of Pharmaceutical Technology, Faculty of Pharmacy, University of Debrecen, Rex Ferenc Street 1, H-4002 Debrecen, Hungary; bacskay.ildiko@pharm.unideb.hu; 3 Department of Industrial Pharmaceutical Technology, Faculty of Pharmacy, University of Debrecen, Rex Ferenc Street 1, H-4002 Debrecen, Hungary; 4 Department of Molecular and Nanopharmaceutics, Faculty of Pharmacy, University of Debrecen, Rex Ferenc Street 1, H-4002 Debrecen, Hungary; rusznyak.agnes@pharm.unideb.hu; 5 Department of Pharmacology, Faculty of Pharmacy, University of Debrecen, Rex Ferenc Street 1, H-4002 Debrecen, Hungary; kajtar.richard@pharm.unideb.hu (R.K.); lekli.istvan@pharm.unideb.hu (I.L.)

**Keywords:** cyclodextrin, absorption, in vitro–in vitro correlation, ex vivo correlation

## Abstract

**Background**: Cyclodextrin (CD) polymers have attracted increasing attention due to their favourable drug delivery properties and broad pharmaceutical applicability. While the bioavailability and biological behaviour of native cyclodextrins have been extensively investigated, considerably less information is available regarding modified cyclodextrin polymers. Therefore, the present study aimed to investigate the permeation and cellular uptake of an epichlorohydrin-crosslinked β-cyclodextrin polymer using multiple in vitro and ex vivo models. **Methods**: Fluorescently labelled β-cyclodextrin polymers were applied in all experiments. Membrane permeation studies were performed using an in-line diffusion cell system with membranes of different pore sizes. In vitro transport and cellular uptake were investigated on HaCaT, Caco-2, and TR146 cell monolayers, while ex vivo permeation studies were carried out using skin, buccal, and intestinal tissues. **Results**: The results demonstrated a strong size-dependent transport behaviour across synthetic membranes. Cell monolayer studies revealed cell-line-dependent differences in polymer intracellular distribution. Lysosomal accumulation was observed in HaCaT and Caco-2 cells, whereas no intracellular accumulation was detected in TR146 cells. These findings suggest differences in polymer permeation among the investigated cell models. Ex vivo studies demonstrated the tissue permeation of cyclodextrin polymers, with marked accumulation within skin layers, indicating predominant dermal retention. Furthermore, strong correlations were identified between the in vitro and ex vivo skin and intestinal models. **Conclusions**: Overall, the findings demonstrate that β-cyclodextrin polymers exhibit complex, barrier-dependent transport behaviour across different biological models. The observed differences in permeation and intracellular localization suggest that multiple transport processes may contribute to their biological interactions, which provide a foundation for future studies aimed at elucidating the molecular mechanisms governing polymer uptake and permeation.

## 1. Introduction

Interest in cyclodextrins and cyclodextrin-based materials has grown significantly in recent decades, as reflected in the PubMed database, where the number of publications has risen steadily since the 1990s when searching for the keyword “cyclodextrin” [1,2,3,4]. Approximately one-quarter of nearly 30,000 publications have appeared in the last five years, indicating an intense increase in research related to cyclodextrin applications [1,4,5]. Scientific interest in cyclodextrin polymers has grown, justified by their favourable physicochemical properties, such as increased material binding capacity, stability, and functional variability [6,7,8,9]. Accordingly, CD polymers have now appeared in a number of industrial and biomedical fields [10], including the food industry [11], environmental technologies, and drug development [2,12,13].

CD-based polymers used in the pharmaceutical industry are primarily used to increase the solubility and bioavailability of active ingredients and to develop controlled drug delivery systems [14,15,16,17,18]. The solubility and biological behaviour of cyclodextrin polymers depend largely on the chemical modifications applied, especially the crosslinking agent [8,19,20]. In pharmaceutical technology, water-soluble polymers are preferred, most of which can be derived from native β-cyclodextrin [15,21]. These polymers include structurally modified forms and polymers with a network structure created with various crosslinking agents, such as epichlorohydrin [19,22]. Our research focuses on the latter category: a commercially available, epichlorohydrin-crosslinked, water-soluble β-cyclodextrin polymer. Cyclodextrin polymer has become the gold standard in polymer research because it is the first reproducibly produced CD polymer whose degree of polymerization and thus molecular size can be standardized, and it consists of an easily performed polymerization step. The polymer contains 50–70% β-cyclodextrin and has an average molecular weight of approximately 50 kDa. Numerous studies have confirmed its applicability in increasing solubility [14,23], enhancing biological stability [17,24], and controlling the release of various active substances [16,18,25], such as indomethacin [26], glipizide [25], and insulin [27,28]. However, no comprehensive study has been conducted to date on the permeation of the polymer by cells and its intracellular fate [29,30].

Therefore, our research aims to investigate the absorption of the gold-standard epichlorohydrin-crosslinked β-cyclodextrin polymer in various in vitro and ex vivo models. Our results may contribute to a deeper understanding of the biological behaviour of CD polymers and provide new guidance for the development of effective cyclodextrin polymer-based drug delivery systems [15,18,31,32].

## 2. Materials and Methods

### 2.1. Materials

The soluble beta-cyclodextrin polymer crosslinked with epichlorohydrin (~50 kDa) (BCDSP) and the fluorescein-labelled βCD soluble polymer crosslinked with epichlorohydrin (FITC-NH-BCDSP) were purchased from Cyclolab Ltd. (Budapest, Hungary). All other reagents were from Sigma-Aldrich Ltd. (Budapest, Hungary).

### 2.2. In Vitro Membrane Permeability of β-Cyclodextrin Polymer

The in vitro permeability of the cyclodextrin polymer was examined using an in-line cell apparatus (In-Line Equilibrium Cell apparatus (Bel-Art Products, Wayne, NJ, USA)). Four membranes with different pore sizes (the 3.5 kDa, 10 kDa, and 50 kDa Spectra/Por 7 cellulose dialysis membranes, and 0.45 µm Teknokroma M.E. cellulose membrane) were used for the measurements. A 10 mg beta-cyclodextrin polymer mixture (containing 90 m/m% BCDSP and 10% FITC-NH-BCDSP as a labelling agent) was dissolved in a phosphate buffer (pH 5.5) to create the standard BCDSP mixture solution as the donor phase. A 500 µL phosphate buffer with a pH of 5.5 was chosen as the acceptor phase. After 30, 60, and 120 min, 200 µL samples were removed from the acceptor phase and replaced with 200 µL of fresh buffer. The cyclodextrin polymer content of the samples was measured by fluorescence spectrophotometry (Fluostar Optima Fluorescence Microplate Reader, from BMG Labtech). The excitation wavelength (λ_exc_) was set to 485 nm, and the emission wavelength (λ_em_) was monitored at 520 nm. The detector gain was set to 2312. Both excitation and emission slit widths were adjusted to 5 nm. All measurements were performed at room temperature (25 ± 1 °C).

### 2.3. In Vitro Cell Monolayer Permeability of β-Cyclodextrin Polymer

For the permeability studies, cell monolayers were grown on 1.12 cm^2^ permeable Transwell^®^ polycarbonate filters (Corning, Lowell, MA, USA) with a pore size of 0.45 µm in 12-well plates. Additionally, 250,000 cells/insert were used to create monolayers. Caco-2, HaCaT, and TR146 monolayers were used for experiments 14–21 days after initial implantation, when the transepithelial electrical resistance (TEER) reached a minimum of 300 Ωcm^2^. The BCDSP mixture was dissolved in Hank’s balanced salt solution (HBSS) at a concentration of 1 m/m %, which, based on our previous studies, did not exhibit cytotoxicity in cell lines [33,34,35,36]. Cell monolayers were washed with HBSS, and 500 µL of the BCDSP mixture solution was applied to the apical surface of the cell layers. Samples were taken after 30, 60, and 120 min from the basal side of the cell layers and replenished with 500 µL fresh HBSS. Finally, TEER values were measured at the end of the experiment to confirm the integrity of the cell monolayers. The cyclodextrin content of the samples was measured by fluorescence spectrophotometry as described in Section 2.2.

### 2.4. Intracellular Distribution of β-Cyclodextrin Polymer on Caco-2, HaCaT, and TR146 Cells

The intracellular distribution of FITC-NH-BCDSP after cellular uptake was evaluated using the following method. In the permeability experiments, Caco-2, HaCaT, and TR146 cells were plated on sterile, round glass coverslips in a 12-well plate (50,000 cells/well). When cells reached the appropriate confluence, samples were washed with Hank’s balanced salt solution (HBSS) and incubated with 500 µL 1 m/m% BCDSP mixture solution for 15, 30, 60, and 120 min at 37 °C in the dark. Then, the samples were washed three times with HBSS and incubated with 50 nM LysoTracker^®^ fluorescent dye (300 µL) for 30 min at 37 °C in the dark. They were then washed three times with HBSS and fixed with a 3.7% paraformaldehyde (PFA) solution for 15 min at room temperature in the dark. Then, cells were washed three times, and cell nuclei were labelled with 283 nM 4′,6-diamidino-2-phenylindole (DAPI) for 10 min at room temperature. At the end of this experiment, the cells were washed again, and the coverslips were mounted to microscope slides. Fluorescence microscopy investigations were conducted with a Zeiss Axioscope A1 (Carl Zeiss AG, Jena, Germany) fluorescence microscope. The following filter sets were used in the microscopy investigations: blue (DAPI)—excitation G 365 nm, emission BP 445/50 nm; green (fluorescein)—excitation BP 470/40 nm, emission BP525/50 nm; red (LysoTracker^®^)—excitation BP 546/12 nm, emission BP 575–640 nm.

### 2.5. Ex Vivo Permeability Study of β-Cyclodextrin Polymer

Ex vivo permeability studies were performed using a Franz diffusion apparatus. Isolated porcine ear and isolated rat intestinal and buccal tissue were used as membranes in the experiment. Ex vivo rat’s tissues were obtained from animals used in a separate study approved by the Hungarian Scientific Ethical Committee on Animal Experimentation (approval No. 7/2023/DEMÁB; approved on 14 December 2023; principal investigator: Dr. István Lekli). No animals were euthanized specifically for the purposes of the present ex vivo experiments. Hank’s balanced salt solution (HBSS) was chosen as the acceptor phase. The stratum corneum of the porcine ear samples was oriented toward the donor compartment. For buccal and intestinal tissues, the epithelial side faced the donor compartment, while the connective tissue side was positioned toward the acceptor compartment. In the donor phase, 500 µL/cm^2^ of a 1 m/m% BCDSP mixture solution was added. During the experiment, samples were taken from the acceptor phase after 30, 60, and 120 min and measured by fluorescence spectrophotometry as described above. In the case of pig ears, the skin layers were separated, and following extraction, the amount of accumulated cyclodextrin polymer was determined using the following method. The pig skin was washed with saline at the application site to remove any remaining polymer solution from the surface. The skin was then dried with cotton wool, and 25 pieces of adhesive cellophane tape were used to peel the skin, thereby removing the subcutaneous layer. The strips were placed in glass vials containing 5 mL of ethanol to extract the cyclodextrin polymer. The remaining skin (epidermis and dermis without the subcutaneous layer) was then heated with a hair dryer for 60 s and separated into epidermis and dermis using a spatula. We cut both parts into small pieces with a scalpel and placed them in 5 mL of ethanol for solvent extraction, which was performed for 24 h and repeated three times.

### 2.6. In Vitro–In Vitro and In Vitro–Ex Vivo Correlations

Correlation analyses were performed to compare permeation across synthetic membranes with different pore sizes (3.5 kDa, 10 kDa, 50 kDa, and 0.45 µm) and epithelial cell monolayer models (HaCaT, Caco-2, and TR146). In vitro–in vitro relationships were evaluated through linear regression analysis, and the strength of correlations was expressed as the coefficient of determination (R^2^). Linear or non-linear relationships were identified depending on the model pair, and relative permeability differences between systems were calculated to describe fold changes in transport. In vitro–ex vivo correlations were assessed between cell monolayers and corresponding tissues (skin, intestinal, and buccal). Linear regression was applied for HaCaT–skin and Caco-2–intestinal comparisons, while buccal tissue data were fitted using a hyperbolic model. Correlation quality was determined using R^2^ values, and kinetic parameters (B_max_ and K_d_) were derived for non-linear relationships.

### 2.7. Statistical Analysis and Modelling

We calculated the apparent permeability coefficient (P_app_) of the cyclodextrin polymer using the following equation:

(1) $$P_{app}=\frac{dQ}{dt}\times \frac{1}{\left(C_{0}\times A\right)}$$

where Papp is the apparent permeability coefficient (cm/s); dQ/dt is the permeability rate of the polymer (mol/s); C_0_ is the initial concentration of the polymer in the donor phase (mol/mL); and A is the membrane area (cm^2^).

The release rate and flux values were calculated using the following equation:

(2) $$Release\;rate\;(J)=\frac{dQ}{A\times dt}$$

(3) $$\mathrm{Flux}\;(J)=\frac{dQ}{A\times dt}$$

where dQ is the change in the amount of polymer released (μg); A is the surface area of the membrane or the membrane onto which the cells were spread (cm^2^); and dt is the change in time (h).

For statistical analysis, GraphPad Prism 6 software (GraphPad Software Inc., San Diego, CA, USA) was used. Dates are presented as means ± SD. Two-way ANOVA with Tukey’s post-test was used in vitro and ex vivo permeability tests. Graphical fitting was performed using linear, second-order polynomial, and hyperbolic regression analysis to evaluate the relationship between the results. A correlation was considered significant when the coefficient of determination (R^2^) exceeded 0.95.

## 3. Results

### 3.1. In Vitro Membrane Permeability of β-Cyclodextrin Polymer

**Figure 1:**
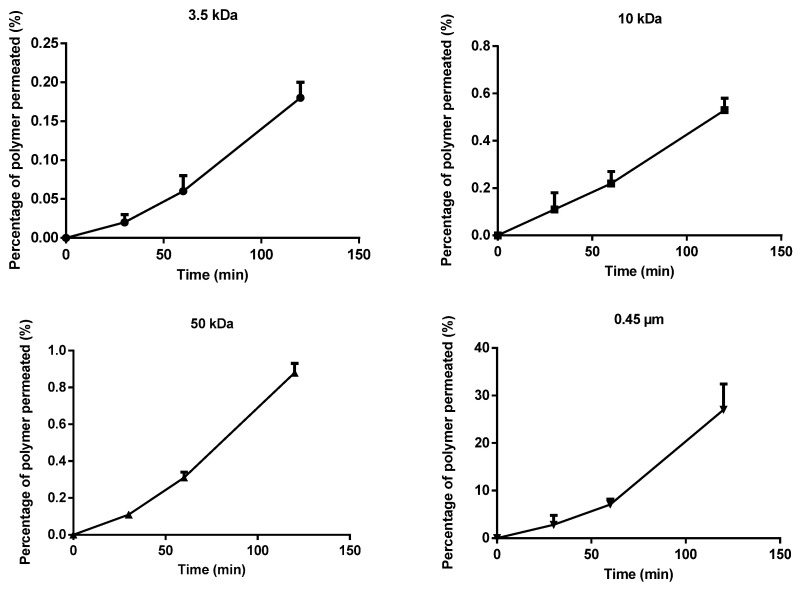
This figure shows the passage of the examined beta-cyclodextrin polymer through membranes with four different pore sizes (3.5 kDa, 10 kDa, 50 kDa, and 0.45 µm) as a function of time. The 3.5 kDa membrane showed significant differences at 30, 60, and 120 min compared to both the 10 kDa and 50 kDa membranes. A significant difference between the 10 kDa and 50 kDa membranes was observed only at 60 and 120 min, *p* < 0.0001. Due to the large difference in size compared to the 0.45 µm membrane, a significant difference is observed for all membranes, *p* < 0.0001.

Membrane permeability experiments were conducted using four different membranes with increasing pore sizes (3.5 kDa, 10 kDa, 50 kDa, and 0.45 µm, based on EMA and FDA recommendations) to evaluate the effect of membrane cutoff on polymer transport. Based on the results (Figure 1), negligible permeation was observed through the 3.5 kDa and 10 kDa membranes, with less than 1% of the polymer crossing these membranes within 2 h. A slight increase in permeability was detected for the 50 kDa membrane, where approximately 1% of the polymer permeated over the same time. In contrast, higher permeation was observed for the 0.45 µm membrane, where nearly 30% of the polymer diffused through the membrane within 2 h, indicating a strong dependence of permeability on membrane pore size.

Quantitative analysis further supported these observations (Table 1). Both the release rate and the apparent permeability coefficient ($P_{app}$) increased progressively with pore size. The lowest values were obtained for the 3.5 kDa membrane. A moderate increase was observed for the 10 kDa and 50 kDa membranes, reflecting limited but measurable transport. A dramatic increase was recorded in the case of the 0.45 µm membrane.

### 3.2. In Vitro Cell Monolayer Permeability of β-Cyclodextrin Polymer

**Figure 2:**
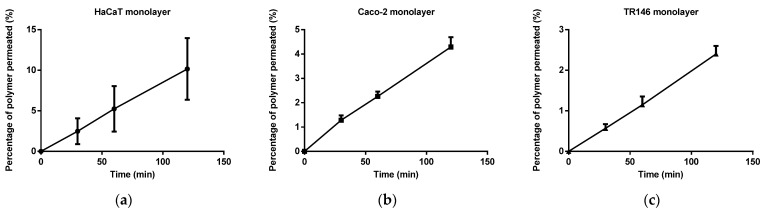
Absorption of beta-cyclodextrin polymer on different cell monolayers as a function of time. The (**a**) shows absorption in HaCaT cell monolayers, the (**c**) shows absorption in Caco-2 cell layers, and the (**b**) shows absorption through TR146 cell monolayers. No significant differences were observed between any of the cell lines after 30 min. When comparing the results from the 60 min samples, the HaCaT cell line showed a significant difference compared to both the Caco-2 and TR146 cell lines, *p* = 0.0008. However, Caco-2 and TR146 did not show a significant difference when compared to each other. For the 120 min samples, a significant difference was also observed for the HaCaT cell line compared to both Caco-2 and TR146, *p* < 0.0001. Caco-2 and TR146 did not show a significant difference even at 120 min.

Our in vitro permeation studies were performed using Franz diffusion cells with HaCaT, Caco-2, and TR146 cell monolayers. TEER values were measured before and after each permeation experiment to assess monolayer integrity, and no significant decrease in the values was observed between these timepoints, indicating that the epithelial barrier remained intact throughout the study. Based on the results of our in vitro absorption study (Figure 2), we found that the lowest amount of polymer, approximately 3%, was permeated through the TR146 monolayer, while slightly more, 4.5%, was permeated through the Caco-2 monolayer in 2 h. The largest amount of polymer, approximately 10%, was permeated through the HaCaT monolayer within 2 h.

As presented in Table 2, the highest permeability was observed in the HaCaT cell model, with a release rate of 224.5 μg/cm^2^·h^−1^ and an apparent permeability coefficient of 12.58 × 10^−6^ cm/s. This indicates enhanced transport across the keratinocyte layer, likely reflecting differences in barrier function and cell-layer integrity. In contrast, the Caco-2 monolayer showed moderate permeability, consistent with its well-characterized tight junction network and established role as a model of the intestinal epithelium. The TR146 model exhibited the lowest permeability, suggesting a more restrictive barrier to polymer transport under the applied experimental conditions.

### 3.3. Intracellular Distribution of β-Cyclodextrin Polymer on Caco-2, HaCaT, and TR146 Cells

**Figure 3:**
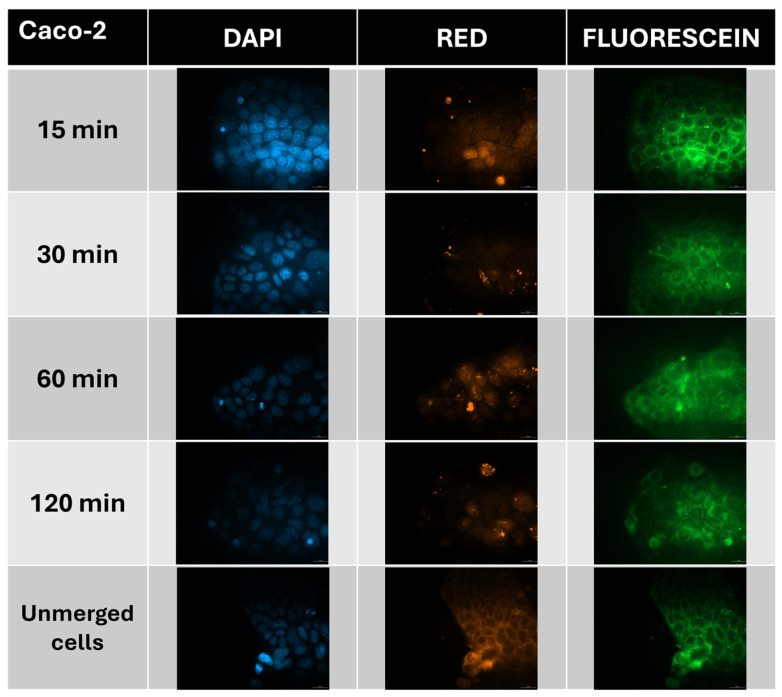
Investigation of the cellular distribution of fluorescently labelled beta-cyclodextrin polymer (FITC-NH-BCDSP) in Caco-2 cells. The labelled cyclodextrin molecule appeared in Caco-2 cells and in vesicles in the cytoplasm in fluorescent microscope images (green pixels—FITC-NH-BCDSP, red pixels—LysoTracker^®^, blue pixels—cell nuclei). Scale bar = 20 µm.

**Figure 4:**
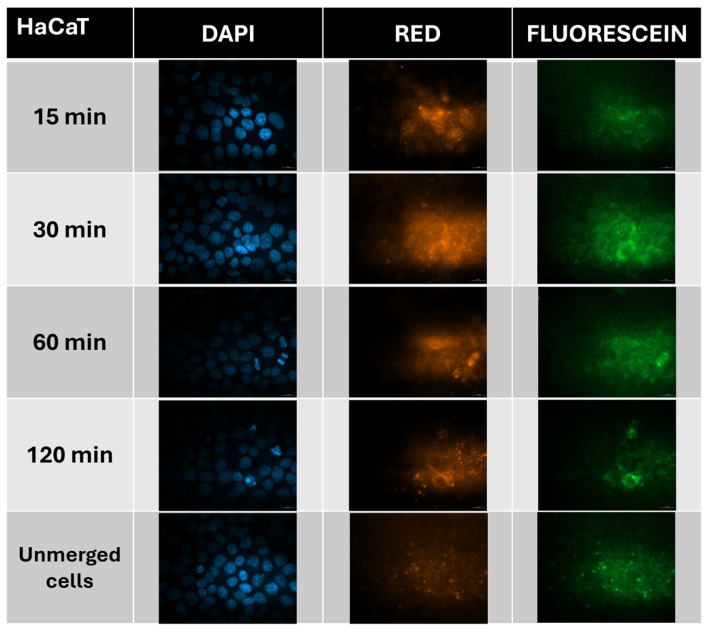
Investigation of the cellular distribution of fluorescently labelled beta-cyclodextrin polymer (FITC-NH-BCDSP) in HaCaT cells. The labelled cyclodextrin molecule appeared in HaCaT cells and in vesicles in the cytoplasm in fluorescent microscope images (green pixels—FITC-NH-BCDSP, red pixels—LysoTracker^®^, blue pixels—cell nuclei). Scale bar = 20 µm.

We examined in vitro the cellular distribution of fluorescently labelled beta-cyclodextrin polymer. The experiments were performed on Caco-2, HaCaT, and TR146 cell lines. For the investigated β-cyclodextrin polymer, a progressive increase in intracellular FITC fluorescence signal was observed over time in the cytoplasm. In HaCaT and Caco-2 cells, the degree of co-localization between the FITC signal and lysosomal markers increased during the incubation period, suggesting progressive accumulation of the polymer within lysosomal compartments at 120 min (Figure 3 and Figure 4).

**Figure 5:**
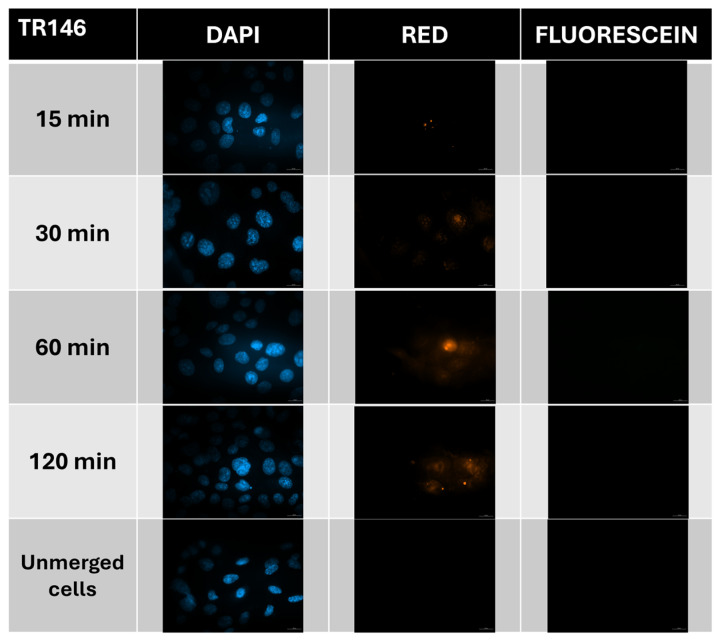
Investigation of the cellular distribution of fluorescently labelled beta-cyclodextrin polymer (FITC-NH-BCDSP) in TR146 cells. The labelled cyclodextrin molecule is not found in the cell cytoplasm nor the cell environment (red pixels—LysoTracker^®^, blue pixels—cell nuclei). Scale bar = 20 µm.

In contrast, in the TR146 cell line, we do not see an increase in the number of lysosomes, only the cell’s own natural number of lysosomes, which can be defined more as background, nor the appearance of fluorescein (Figure 5). The absence of detectable lysosomal localization may indicate that the polymer is transported across the epithelial barrier without substantial intracellular retention.

### 3.4. Ex Vivo Permeability Study of β-Cyclodextrin Polymer

**Figure 6:**
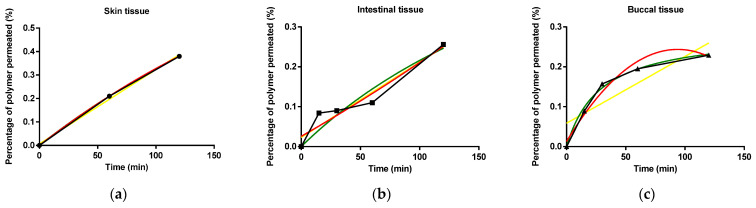
Results of our ex vivo absorption experiment as a function of time. The (**a**) shows absorption in pig ear skin tissue, the (**c**) shows absorption in rat intestinal tissue, and the (**b**) shows absorption of FITC-NH-BCDSP in rat buccal tissue. In the case of fitting, the red line indicates a second-order polynomial fit, the green line indicates a hyperbolic fit, and the yellow line indicates a linear fit.

We conducted our ex vivo permeability tests on rat intestinal tissue, rat oral mucosa tissue, and pig ear skin tissue. Based on our permeability tests, the polymer permeated through the oral mucosa and intestinal tissues in almost equal amounts, approximately 0.25%, within 2 h. In the case of skin, 0.4% of the polymer was permeated within 2 h. Absorption through skin tissue was uniform, while absorption through intestinal and buccal tissues was not completely linear (Figure 6). However, in the case of buccal tissue, a saturation phase can be observed, after which the rate of absorption slows down, resulting in a hyperbolic curve. The correlation with different kinetics shows the following results: in the case of skin tissue, linear fitting was observed (R^2^ = 0.9922). For intestinal tissue, the best fit was provided by the second-order polynomial (R^2^ = 0.9300). For buccal tissue, the hyperbolic function provided the most accurate fit (R^2^ = 0.9939).

As summarized in Table 3, the highest flux (J) was observed in skin tissue, while the corresponding P_app_ remained relatively low. In contrast, intestinal tissue exhibited a lower flux but a higher P_app_ value, suggesting more efficient permeation relative to applied concentration. The lowest flux was measured in buccal tissue, accompanied by a moderate permeability coefficient, indicating a comparatively restrictive barrier under the applied experimental conditions.

**Figure 7:**
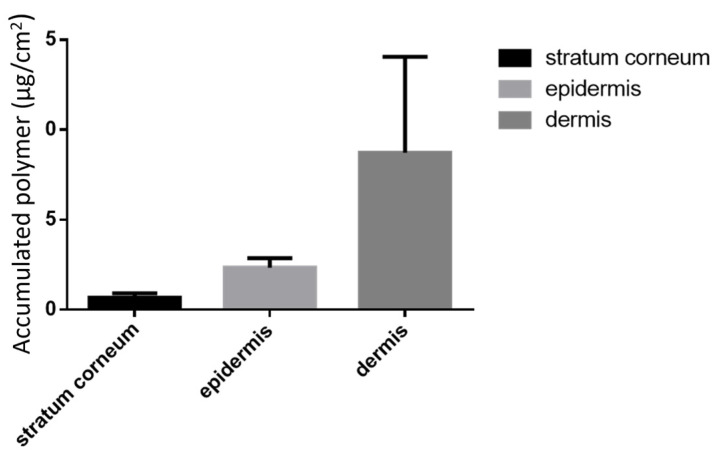
Polymer accumulation in the skin between different layers of skin tissue.

After evaluating transdermal absorption, we further investigated skin retention. Owing to the complex skin structure, effective delivery often requires not only permeation but also accumulation and storage within specific layers. Therefore, we examined whether the cyclodextrin polymer can accumulate in different layers of the skin. Our results showed that the polymer accumulated in different layers of the skin (Figure 7), with the highest concentration found in the dermis. Much smaller amounts of polymer remained in the stratum corneum and epidermis. Based on these findings, we conclude that the polymer is continuously absorbed into the skin tissue, as evidenced by the polymer recovery results shown in the previous figure, but complete absorption takes much longer than the duration of the ex vivo experiments.

### 3.5. In Vitro–In Vitro and In Vitro–Ex Vivo Correlation

#### 3.5.1. In Vitro Membrane–In Vitro Cell Correlation

**Figure 8:**
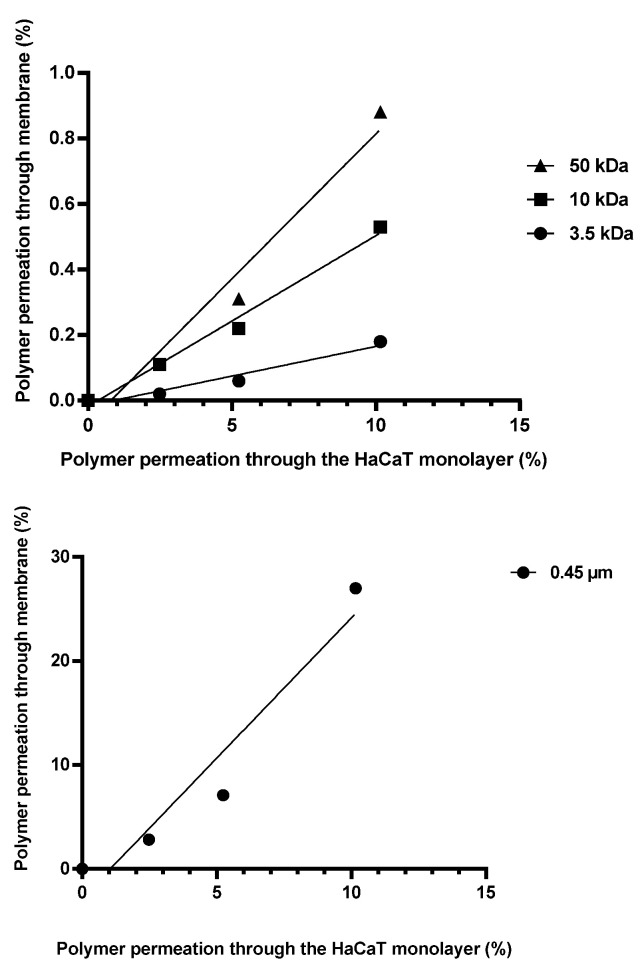
In vitro membrane–in vitro HaCat cell line correlation (the different pore sizes are indicated in the figures).

Correlation analysis between absorption on the HaCaT cell line and permeation across membranes with different pore sizes (Figure 8) revealed a linear relationship for the 3.5 kDa (R^2^ = 0.957), 10 kDa (R^2^ = 0.988), and 50 kDa (R^2^ = 0.965) membranes. The amount of polymer permeating through the HaCaT monolayer was approximately 50-fold higher than that passing through the 3.5 kDa synthetic membrane within the same time. A similar trend was observed for the 10 kDa membrane, where the amount of polymer transported across the HaCaT cell layer was approximately 25-fold greater. In the case of the 50 kDa membrane, the difference was reduced to approximately 10-fold. Comparing the HaCaT monolayer and the membrane with a pore diameter of 0.45 µm, we can see almost identical absorption rates for the polymer, but the two in vitro methods do not show a linear correlation (R^2^ = 0.927).

**Figure 9:**
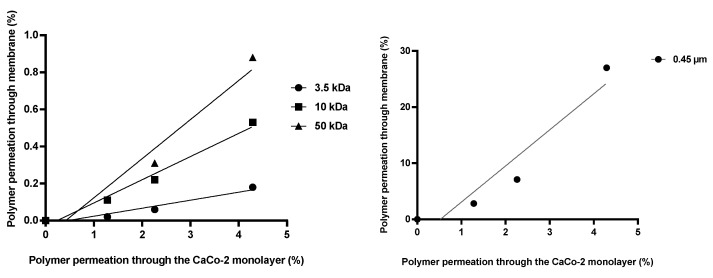
In vitro membrane–in vitro Caco-2 cell line correlation (the different pore sizes are indicated in the figures).

Comparing the absorption on the Caco-2 cell line and on membranes with different pore sizes (Figure 9) revealed linear relationships for the 10 kDa membrane (R^2^ = 0.981). The amount of active compound permeating through the cell monolayer was approximately 10-fold higher than that passing through the membrane. Nonlinear correlations were detected in the case of the 3.5 kDa (R^2^ = 0.940), 50 kDa, and 0.45 µm (R^2^ = 0.911) pore-sized membranes.

**Figure 10:**
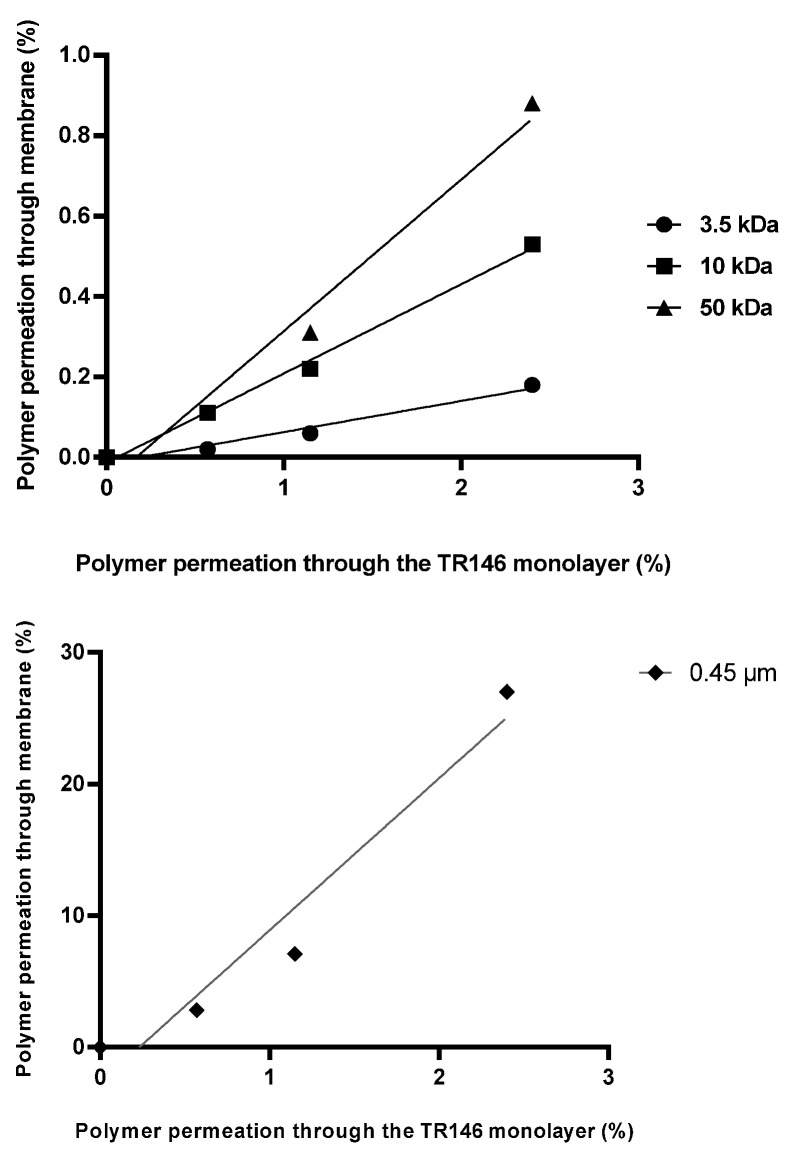
In vitro membrane–in vitro TR146 cell line correlation. (The different pore sizes are indicated in the figures.).

Linear relationships were observed between the TR146 cell line and the 3.5 kDa (R^2^ = 0.969), 10 kDa (R^2^ = 0.995), and 50 kDa (R^2^ = 0.976) membranes (Figure 10). The amount of polymer permeating through the TR146 monolayer within the same time was approximately 10-fold higher than that passing through the 3.5 kDa synthetic membrane. For the 10 kDa membrane, the permeated amount was approximately 5-fold higher, while the 50 kDa membrane was approximately 3-fold higher. No linear relationship was observed with the 0.45 µm (R^2^ = 0.945) pore-sized membrane.

#### 3.5.2. In Vitro–Ex Vivo Comparison

**Figure 11:**
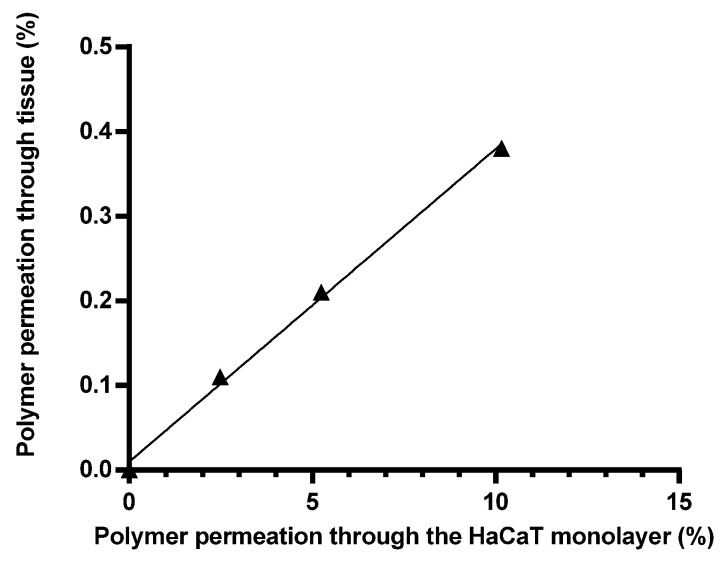
In vitro HaCaT cell line monolayer–ex vivo skin tissue correlation.

The in vitro and ex vivo comparisons were performed between tissues and cell monolayers. First, we analyzed the skin tissue correlation with the HaCaT cell line, the results of which are shown in Figure 11. The linear R^2^ value was 0.9969, and the relationship was described by the equation y = 0.03699 × x + 0.009663.

**Figure 12:**
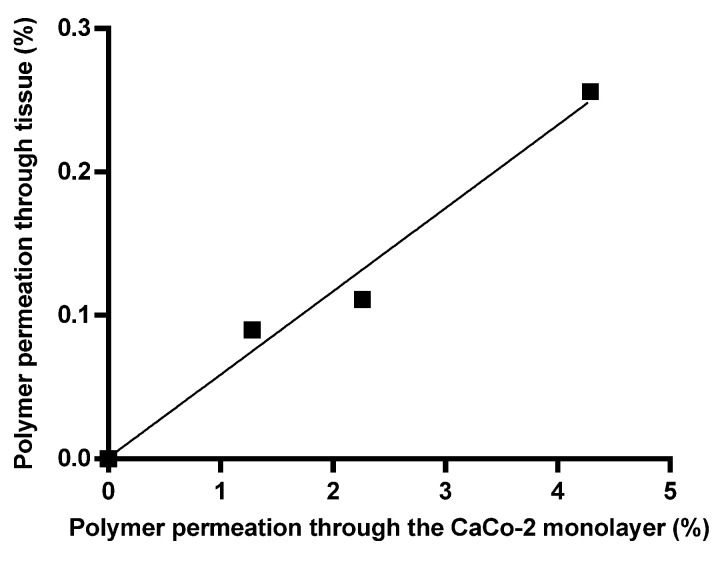
In vitro Caco-2 cell line monolayer–ex vivo intestinal tissue correlation.

In the case of the intestinal correlation (Figure 12), a linear correlation can be found between the cell line absorption and tissue penetration, and this relationship can be described by the equation y = 0.05800 × x + 0.0007158 (the R^2^ value was 0.9792).

**Figure 13:**
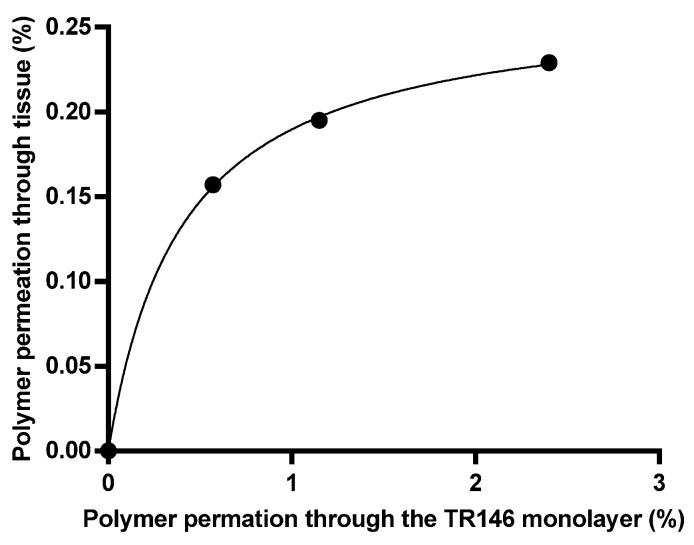
In vitro TR146 cell line monolayer–ex vivo buccal tissue correlation.

Finally, we analyzed the buccal correlation based on the results (Figure 13), and we obtained a hyperbolic kinetic. B_max_ = 0.2663 and K_d_ = 0.4040 were found, so the equation that can be used for the calculation is y = (0.2663 × x)/(0.4040 + x).

## 4. Discussion

This study provides a comprehensive overview of the permeability of epichlorohydrin-crosslinked β-cyclodextrin polymers and their fate at the cellular level through synthetic membranes, epithelial cell layers, and biological tissues, with a particular focus on transport processes in different barrier systems.

In our in vitro membrane permeability experiments, we chose 3.5 kDa and 10 kDa membranes because these have already been used in numerous studies investigating the in vitro absorption of various nanoparticle materials or polymers [37,38,39,40]. The use of the 50 kDa membrane was justified by the molecular size of the cyclodextrin polymer employed in this study, and similar membranes have also been frequently utilized in absorption-related research [41,42]. In addition, the 0.45 μm pore-sized membrane was chosen because it represents one of the most commonly used membrane types in studies of transdermal drug delivery systems [43,44,45]. The results obtained in our experiments clearly demonstrated size-dependent transport behaviour. Less than 1% of the polymer passed through membranes with pore sizes of 3.5 kDa and 10 kDa within 2 h, while only ~1% permeation was observed for the 50 kDa membrane. This result is consistent with previous observations that the effective hydrodynamic size of crosslinked cyclodextrin polymers exceeds their nominal molecular weight due to branching and the formation of a hydration shell [46,47]. Similarly, limited permeability has been reported for cyclodextrin-based nanosponge and polymer drug delivery systems, where diffusion through dialysis membranes is primarily determined by steric hindrance rather than molecular weight alone [32]. The significantly higher (~30%) permeation observed on the 0.45 µm pore-sized membrane confirms that, when using macroporous membranes, transport is controlled by pore geometry rather than molecular diffusion. This behaviour is consistent with the results of transdermal diffusion studies, which show that macroporous membranes allow convective transport of polymer systems [48,49,50].

For our in vitro cell monolayer studies, we chose the HaCaT cell line because it is stable, highly reproducible, and the gold standard for in vitro skin absorption studies [51,52]. We selected the Caco-2 cell line for similar reasons, as it is most commonly used for in vitro modelling of intestinal absorption [53,54,55]. The TR146 cell line is used to model absorption in the oral mucosa and, like the others, is stable and highly reproducible [56]. Interestingly, based on the experimental results, the biological barriers allowed for significantly greater transport than the synthetic membranes. For example, approximately 10% of the polymer passed through the HaCaT cell layer in 2 h compared to <1% permeation measured on the 3.5 kDa membrane. This difference supports previous observations that epithelial models allow active or facilitated uptake mechanisms in addition to passive diffusion [57]. It is known that keratinocytes can internalize macromolecules via endocytosis, which may explain the increased transport observed in the HaCaT model [58]. The ~4.5% transport measured on the Caco-2 cell layer also fits well with the literature data describing moderate permeability in cyclodextrin-based systems in intestinal epithelial models [59,60]. The lower transport (~3%) detected on the TR146 cell layer reflects the well-known barrier function of the oral mucosa against hydrophilic macromolecules [61].

Fluorescence imaging revealed cell-type-specific patterns, and our measurements also showed that two of the three cell lines, namely, the HaCaT and Caco-2 cell lines, exhibited stronger autofluorescence, a finding that had been demonstrated previously [62,63]. In addition, progressive co-localization of the polymer-derived FITC signal with lysosomal markers was observed in HaCaT and Caco-2 cells, indicating intracellular accumulation within lysosomal compartments over time [64]. It is known that cyclodextrins and their derivatives enter epithelial cells via vesicular pathways and accumulate in lysosomes prior to cytoplasmic redistribution [65,66]. A similar mechanism has previously been described for β-cyclodextrin derivatives in intestinal cells [67]. In contrast, no intracellular accumulation was detected in TR146 cells, despite measurable permeation through the cell layer. This suggests that transport may occur via paracellular pathways or rapid transcellular passage without lysosomal storage. A similar phenomenon has been described for hydrophilic macromolecules in mucosal epithelial systems [68]. These observations suggest that polymer transport and intracellular processing differ among the investigated epithelial cell models. However, the specific cell-surface proteins, uptake pathways, and dominant transport mechanisms were not investigated in the present study and therefore remain to be elucidated in future work.

We chose porcine skin tissue for our ex vivo measurements because numerous ex vivo and in vivo experiments have already confirmed that there is a close correlation with absorption in human tissues [69,70]. For the intestinal and oral mucosa models, we chose the rat model because it is readily available and has long been used for ex vivo studies of oral formulations and oral administration [71,72,73,74]. The ex vivo studies further confirmed the limited but measurable transport of the polymer. Permeation of approximately 0.25% was observed in intestinal and buccal tissues, while ~0.4% permeated skin within 2 h. These values are consistent with previously reported data on the permeability of hydrophilic polymer drug carriers through biological barrier systems [75]. The slightly higher skin permeation is consistent with studies that have confirmed the predictive significance of pig skin in modelling human dermal absorption [76,77].

It is important to note that polymer accumulation in the dermis suggests depot formation [78,79] rather than rapid systemic penetration. Similar dermal retention has been described for other polymeric drug delivery systems, which is often associated with the possibility of sustained drug release [80].

Comparison of the in vitro and ex vivo systems revealed a strong linear correlation in both the skin and intestinal models. Similar predictive relationships between epithelial cell models and biological tissues have previously been reported in permeability studies of nanocarrier systems [81]. In contrast, the hyperbolic behaviour observed in the buccal model suggests saturation kinetics, which is characteristic of carrier-limited or biologically mediated transport processes and has been described previously during the permeation of macromolecules across mucosal membranes [82].

Overall, the results show that the transport of the β-cyclodextrin polymer studied occurs in a complex manner in the case of synthetic barriers, characterized by size-limited diffusion. Epithelial cell models showed cell-type-dependent differences in intracellular localization and permeation. The ex vivo experiments further confirmed the limited but measurable transport of the β-cyclodextrin polymer across biological tissues. In skin tissue, accumulation can be observed. Together, these findings highlight the complex biological interactions of crosslinked β-cyclodextrin polymers and support their further investigation as promising carrier materials for drug delivery applications [5,83,84].

## 5. Conclusions

In conclusion, the investigated β-cyclodextrin polymer exhibited barrier-dependent transport behaviour. While permeation through synthetic membranes was strongly limited by size-dependent diffusion, the biological models showed enhanced transport accompanied by differences in intracellular localization and polymer–cell interactions. The precise uptake pathways and the molecular mechanisms underlying these cellular differences remain to be elucidated in future studies. The polymer also demonstrated dermal accumulation, suggesting potential depot formation. Overall, these findings indicate that crosslinked β-cyclodextrin polymers interact with biological barriers rather than behaving as inert excipients.

## Figures and Tables

**Table 1:** This table lists the release rates and P_app_ values typical for membranes.

Membrane Pore Size	Release Rate (μg/cm^2^ × h^−1^)	P_app_ (×10^−7^ cm/s)
3.5 kDa	11.46 ± 1.00	3.18 ± 0.27
10 kDa	33.75 ± 3.34	9.37 ± 0.92
50 kDa	56.17 ± 3.16	15.60 ± 0.87
0.45 µm	1721.86 ± 347.13	478.29 ± 96.42

**Table 2:** This table lists the release rates and P_app_ values which are characteristic of the various cell monolayers.

Cell Monolayer	Release Rate (μg/cm^2^ × h^−1^)	P_app_ (×10^−6^ cm/s)
HaCaT	224.5 ± 94.53	12.58 ± 4.70
Caco-2	120.09 ± 65.23	5.33 ± 0.48
TR146	73.21 ± 30.08	2.96 ± 0.26

**Table 3:** This table lists the flux and P_app_ values for various tissues.

Tissue Type	Flux J (μg/cm^2^ × h^−1^)	P_app_ (×10^−6^ cm/s)
skin tissue	9.53 ± 0.49	0.53 ± 0.02
intestinal tissue	5.87 ± 1.26	1.36 ± 0.24
buccal tissue	2.02 ± 0.11	0.87 ± 0.75

## Data Availability

The data that support the findings of this study are available from the corresponding author upon reasonable request.

## Abbreviations

The following abbreviations are used in this manuscript:

CD	Cyclodextrin
βCD	Beta-cyclodextrin
HaCaT	Immortalized cell line of human skin keratinocyte cells
Caco-2	Immortalized cell line of human colorectal adenocarcinoma cells
TR146	Immortalized cell line of human buccal mucosa cells
BCDSP	Soluble beta-cyclodextrin polymer crosslinked with epichlorohydrin
FITC-NH-BCDSP	Fluorescein-labelled βCD soluble polymer crosslinked with epichlorohydrin
TEER	Transepithelial electrical resistance
HBSS	Hank’s balanced salt solution
PFA	Paraformaldehyde
DAPI	4′,6-diamidino-2-phenylindole
